# SCB-HC-ECC–Based Privacy Safeguard Protocol for Secure Cloud Storage of Smart Card–Based Health Care System

**DOI:** 10.3389/fpubh.2021.688399

**Published:** 2021-09-30

**Authors:** Sudha Senthilkumar, K. Brindha, Natalia Kryvinska, Sweta Bhattacharya, Giridhar Reddy Bojja

**Affiliations:** ^1^School of Computer Science and Engineering, Vellore Institute of Technology, Vellore, India; ^2^School of Information Technology and Engineering, Vellore Institute of Technology, Vellore, India; ^3^Head of Information Systems Department, Comenius University, Bratislava, Slovakia; ^4^School of Information Technology and Engineering, Vellore Institute of Technology, Vellore, India; ^5^College of Business and Information Systems, Dakota State University, Madison, SD, United States

**Keywords:** user anonymity, ECC, MAT tree, health care information, smart card

## Abstract

The advent of the internet has brought an era of unprecedented connectivity between networked devices, making one distributed computing, called cloud computing, and popular. This has also resulted in a dire need for remote authentication schemes for transferring files of a sensitive nature, especially health-related information between patients, smart health cards, and cloud servers *via* smart health card solution providers. In this article, we elaborate on our proposed approach for such a system and accomplish an informal analysis to demonstrate the claim that this scheme provides sufficient security while maintaining usability.

## Introduction

With the advent of cloud computing, we can rent servers and run geophysical modeling applications on the authoritative node present everywhere globally. We can securely store an enormous amount of data that can be accessed only by authorized users and applications (1–3). It enables us to rent a virtual server, switch it on or off, and expand it to fulfill users' immediate requirements. It increases association, adaptability, availability, and competency and speeds up the development process to imitate the deviations afforded to workload demand and also provides cost reduction over with efficient and optimized computations (4–8). As healthcare evolves, the need for innovative information system development is necessary (9, 10).

Cloud computing is the new paradigm for outdated conventional computing by adopting newer technology and many economic aspects. It is beneficial for both customers and service providers (2, 11). However, it has many advantages and disadvantages that restrict its usability. It includes architecture that supports many potential applications, programming models to support vast-scale data-centric computing, and provision for security and privacy protection of data. Security of data is challenged by both outside and inside threats (12–14). They can make use of a user's data for their benefit. Consequently, the popularity of cloud computing increases issues in security and privacy areas as well (15–17).

Consider a healthcare organization in which patients use a smart card that electronically holds patients' medical information. A smart card mechanism is used globally for secure identity, access, and payment applications. Smart health card solutions for patient and provider identity management are deployed worldwide and are accessible from various vendors (18, 19). A smart card mechanism offers a robust foundation for healthcare ID cards, empowering enhancement in healthcare procedures and in-patient and provider identity verification while securing data and protecting privacy (20–22). Smart healthcare cards are available with two chips, one for the patient, and one for health professionals. Smart health cards can be an essential information source in case of an emergency when the patient is unresponsive. It could be the first source of information to know about the patient. However, smart cards are restricted in memory size, allowing the storage of only a limited amount of data. As such, the memory-intensive data, such as lab reports or diagnostic images and additional patient-related information, can be stored in a cloud server and accessed *via* the smart health card by healthcare professionals through the smart card solution provider (23–25).

Moving over to the cloud has proven to be helpful for both healthcare professionals and patients. The cloud also engages the patient with their health insurance plans by offering them generous access to their additional healthcare data that is not there in the smart health card, resulting in improved patient outcomes. Providing health care data in the cloud engages the interoperability of several segments of the health care industry, such as pharmaceuticals, insurance, and payments (13, 26, 27).

The following sections of the paper are organized as follows. Section II discusses related works on implementing and authentication of a smart card–based health care system with the cloud. Section III gives the preliminaries required for our work. Section IV gives an overview of the proposed system and system model. Section V describes the security and performance analysis of our scheme compared with other schemes. The conclusion is provided in Section VI.

## Related Works

Many works have addressed implementing and authenticating smart card–based health care information systems. Moudgil et al. (1) designed a cloud-based smart health card monitoring system. Their proposed monitoring system helps health care providers, such as hospitals, physicians, and pharmacists, by managing all the patient data electronically, securely, and efficiently. It uses Bluetooth technology to transmit live patient monitoring data. It also supports off-line storage of medical and information and periodic updates to the cloud database. However, they do not focus on how the smart card and cloud servers are synchronized and how the mutual authentication happens between them. Yang et al. (28) design a MedShare system that publishes patient data to a cloud server using a two-way authorization process. They use the national identification card that patients swipe to publish data in the cloud. However, identification cards are only used for authentication purposes and do not carry any health care information. Li et al. (29) design a mutual authentication and privacy preservation protocol for the TMIS system. They use the AES encryption algorithm for encrypting patient information. Kausar et al. (30) design an intelligent card-based system using an iris-based biometric cryptosystem for an innovative card-based healthcare system. They focus only on how the patient data is stored and retrieved in the smart card. Their system does not include any security phases and is not integrated with cloud storage (31, 32).

Al-Saggaf et al. (33) propose a biometric-based remote authentication scheme using a smart card. They use a hashing function for transferring all the information. However, they do not mention the specific cryptographic technique for storing the data in the smart card. Kumari et al. (23) design an ESEAP system, which is an ECC-based mutual authentication protocol for the smart card. However, their system does not support the various phases, including health center data upload, medical data upload, and the lab technician phase. Ganesh et al. (34) propose the smart, automated health machine using IoT, which provides health services to the local area. They discuss the authentication phase using the smart card system to secure their privacy, but the system is not integrated with the cloud (35, 36).

The research work emphasizes the authentication to recognize that unauthorized users cannot access a user's private data but disregards an elusive privacy issue. In contrast, data sharing happens between the other users, such as the patient's smart card medical data and the cloud service provider. We propose a solution to address the data-sharing privacy issue for this type of environment.

The main contribution of the article is as follows:

1. Mutual access authority is attained by an anonymous access request matching approach with concern about security and privacy so that the cloud is not aware of who the patient is.
2. Mutual authentication between the healthcare organization that accesses the patient data using a smart card *via* the smart health card solution provider to the cloud server for further treatment.
3. The ECC-based encryption on the patient-related data in the cloud server and the smart health card.
4. Notification to the smart health card solution provider about changes in the patient data by the health care organization.

## Preliminaries

### Elliptic Curve Cryptography

**Elliptic-curve cryptography** (**ECC**) is a technique for an asymmetric cryptosystem built on the algebraic structure of elliptic curves over finite fields (29).

Let *p* be a large prime number and E denote the elliptic curve over the prime finite field *Z*_*P*_


$$\begin{array}{l} E:\;y^{2}=\;x^{3}+c.x+d\;\left(mod\;p\right)\text{with}\left(\text{c},\text{d}\right)\in \;Z_{P}\text{ and }4c^{3}\\ +27d^{2}\left(mod\;p\right)\#0 \end{array}$$

and produces grouping


$$\begin{array}{l} E_{p}\;\left(c,d\right). \end{array}$$

Base point G on the elliptic curve *has a large order* *n*, where *n* is a large prime number.

#### Encryption

1. Encode the message as (x,y) of point P_m_ *m* → *P*_*m*_:(*x, y*) on the ECC
2. Generate (pub,priv) key pair to be generated
3. Let k be the random number such as positive integer selected by A

$$\begin{array}{l} C_{m}=\left(KG,P_{m}+kP_{B}\right) \end{array}$$

where
*P*_*m*_ is the plain text point and *P*_*B*_ = *n*_*B*_ * *G*
*where*_*n*_*B*_ < *n*_*which is private key*, *P*_*B*_ is the public key and *C*_*m*_ is the cipher text.

**Table:** 

**References**	**Method**	**Metrics**	**Limitation/research challenge**
Moudgil et al. (1)	Smart card–based integrated electronic health record system	Biomedical parameters, such as blood pressure, diabetes mellitus and pulse oxygen.	Not focused on how the smart card and cloud servers are synchronized with each other and how the mutual authentication happens between them
Yang et al. (28)	MedShare system that uses two-way authorization techniques	The various phases are measured with respect to response time in milliseconds, throughput in bits/second and bandwidth in KB/second	Identification card only used for authentication purposes; does not carry any health care information
Li et al. (29)	Mutual authentication and privacy preservation protocol for TMIS system	Total cost of healthcare center upload phase, patient data upload phase, treatment and checkup phases are measured in seconds	Asymmetric encryption technique not used for encrypting the data
Kausar et al. (30)	A smart card–based system using an iris-based biometric cryptosystem for smart card–based healthcare system	Measurement of false rejection rate and false acceptance rate	They focus only on how the patient data is stored and retrieved in the smart card. The system does not include any security phases and is not integrated with cloud storage.
Al-Saggaf et al. (33)	Collision-resistant hash method	Computational cost of login and registration phases are measured in milliseconds	They use a hashing function for transferring all the information; however, they do not mention the specific cryptographic technique for storing the data in the smart card.
Kumari et al. (23)	ESEAP system that is an ECC-based mutual authentication protocol for smart cards	Communication cost measured in seconds	System does not support the various phases, which include health center data upload, mediclaim data upload, and Lab technician phase.
Divya et al. (34)	Smart automated health machine using IoT	Measurement of human heart rate, blood pressure, and ECG	Not focused on system integration with cloud
Sanjuan et al. (35)	Message queuing telemetry transport protocol using cryptographic smart card	Time spent for cryptographic operations measured in milliseconds	System performance is to be improved by using ECC algorithm instead of RSA.

#### Decryption

1. Let us find *p*_*m*_ = *p*_*m*_ + *KP*_*B*_ − *n*_*B*_ * *kG*
2.
$KP_{B}=k\left(n_{\begin{array}{c} B*\\ \; \end{array}}G\right)=kn_{B}\;*\;kG$

3. Because of the multiplicative inverse property, *kn*_*B*_ * *kG* can be written as *n*_*B*_ * *kG*
4. *p*_*m*_ = *p*_*m*_ + *n*_*B*_ * *kG* − *n*_*B*_ * *kG*

Finding the value of k or private key *n*_*B*_ is an elliptic curve discrete logarithmic problem (ECDLP) that requires a fully exponential running time. To compute the 160-bit key size of private key *n*_*B*_, we require 8.5 ^*^ 10^11^ MIPS.

### ECCDSA

The elliptic curve equivalent of the digital signature algorithm (DSA) is the elliptic curve digital signature algorithm (ECDSA). The ECDSA was first projected in 1992 by Scott Vanstone in response to NIST (37).

ECDSA has three phases: key generation, signature generation, and signature verification.

ECDSA Key Generation:

A is an entity that uses the key pair with a particular set of ECC domain parameters (p,q,g) that does the following:

1. Choose the pseudo random integer d in the interval 1 ≤ *d* ≤ *q* − 1
2. Calculate *P* = *dG*
3. Choose P as its public key, and d is the private key

ECDSA Signature generation

A's message m is signed with domain parameters D = {*q, FR, a, b, G, n*.*h*} and key pairs (P,d) perform the following steps:

1. Choose a random integer z, 1 ≤ *x* ≤ *n* − 1
2. Calculate xG = (*x*_1_,*y*_1_) and change *x*1 to an integer ${\bar{x}}_{1}$
3. Calculate *s* = *x*_1_*mod n*. If s=0, then go to step 1
4. Calculate *z*^−1^*mod n*.
5. Calculate SHA-1(m) and convert the output to an integer f.
6. Calculate v = *z*^−1^(f+ds) mod n. If v=0, then go to step 1.
7. A's signature for the message m is (s,v).

ECDSA Signature verification

To verify A's signature (s,v) on m, the entity B gets an authentic copy of A's domain parameters D = {*q, FR, a, b, G, n*.*h*} and its public key P. B does the following:

1. Verify (s,v) are integers in the interval [1,n-1]
2. Calculate SHA-1 (m) and convert the output to an integer f
3. Calculate w = v^−1^mod n
4. Calculate i_1_=fw mod n and i_2_=sw mod n
5. Calculate X = i_1_G + i_2_Q
6. Calculate X = 0; then reject the signature. Otherwise, convert x coordinate *x*1 of X to an integer and then ${\bar{x}}_{1}$ calculate u= ${\bar{x}}_{1}$ mod n
7. Accept the signature only if u = s

### SHA-256

Secure hash algorithm-256 (SHA-256) is a cryptographic hash function with a message digest size of 256 bits. It is a keyless hash function; it detects the changes in the message called the manipulation detection code (MDC). A message is handled by blocks of 512 = 16 × 32 bits, in which each block is needful of 64 rounds (38–40).

It uses the Boolean operations AND, XOR, OR, and Bitwise complement that are indicated by ∧, ⊕ and ∨, ^−^. Integer addition modulo 2^32^, indicated by A + B.

The *RotR(A, m)* indicates the circular right shift of m bits of the binary word A.

The *ShR(A, m)* indicates the right shift of m bits of the binary word A.

*A||B* denotes the concatenation of the binary words A and B.

## System Model

### Architecture

**Figure 1:**
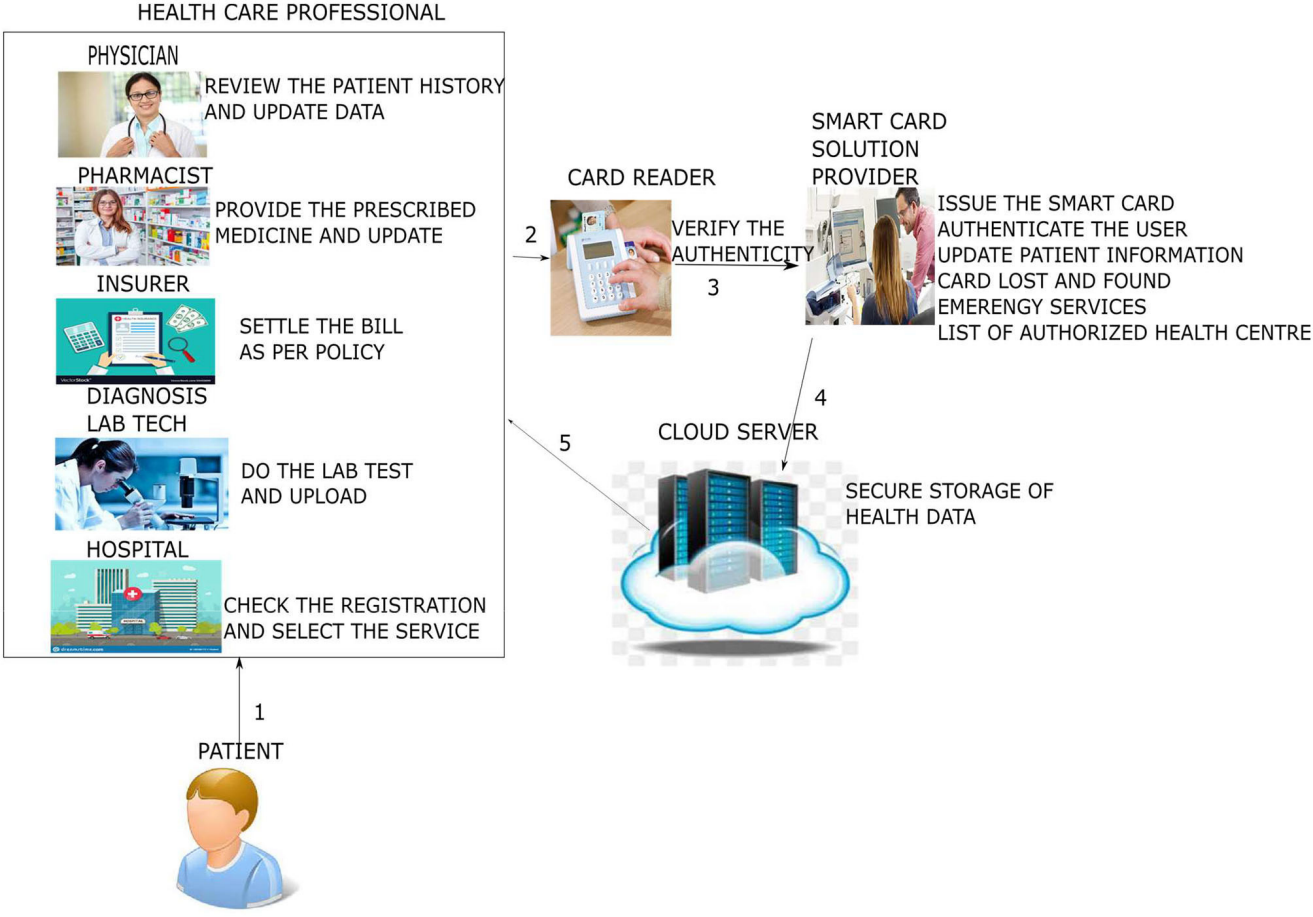
System architecture.

The proposed system emphasizes the elimination of all the above stated factors that are discussed in the existing systems (23, 41, 42). The architecture of the proposed system is shown in Figure 1.

The system architecture shows that the patient goes to the health care professional, such as doctors or pharmacists, and health insurers and diagnosis lab technicians show how the interactions occur. First, both the patient and health care professional swipe their respective smart cards in the card reader: the patient's smart card by the patient and the professional smart card by the health care professional to mutually authenticate each other. Subsequently, the patient performs the second-factor authentication by entering a PIN or password or biometric. After the authentication phase, the patient's basic medical pieces of information are read from the patient's smart card. Suppose further detailed medical information is necessary to proceed with the next level, such as to take treatment from the doctor, to purchase medicine from the pharmacist, or to claim the insurance from the health insurer. In that case, the cloud server is contacted *via* the smart card solution provider (19, 43–45). The cloud server responds to the health professional's request by fetching the patient information and sending it to the health care professional. Later, when the patient data are modified or additional information needs to be added, the data are sent to the healthcare professional's cloud server. The following are the various phases of our proposed protocol.

### Adversary Model

In this paper, we regard the adversarial model as follows:

1. X to capture the message transmitted on the cloud environment (46, 47).
2. The security parameters present in the smart card can be extracted by X (36, 48).
3. The password dictionary can be computed by X off-line (33, 48).

**Table:** 

**Notations**	**Description**
*U_*i*_*	User i
X	Adversary
*R*	Nonce
*pwd_*i*_*	Password for user i
*e_*i*_*	E-mail for user i
P	Elliptic curve base point
*h*(·)	SHA-256 hash function
*M_*R*_*	User registration message
S	Nonce $\in Z_{P^{*}}$
Y	Public key
A	Nonce
T	Current time stamp
Δ*T*	Maximum transmission delay

#### Preliminary Phase

When the smart card is purchased from the smart card issuer, the patient's basic personal information or health care professional's information is stored in an encrypted form using ECC. To do that, the following ECC security parameters are chosen.

In this phase, the cryptographic algorithm ECC is chosen by the health care professional as well as by the smart card solution provider (SP) for encrypting the patient data in the patient's smart card and the detailed information of the patient's medical information stored in the cloud server.

#### Algorithm for Preliminary Phase

Input: ECC parameters

Output: Public key Y and private key s

**1:** Let p be a large prime number, and E denote the elliptic curve over the prime finite field *Z*_*P*_.

**2:** Smart card SP chooses an ECC, *E*:*y*^2^ = *x*^3^ + *c*.*x* + *d* (*mod p*) and generates grouping *E*_*p*_ (*c, d*) *having order* *n*, where *n* is a large prime number.

**3:** Then the smart card SP chooses base point,

*P* = (*x*_0_, *y*_0_), where *n*.*P* = *O* Later,

**4:** SP picks a nonce $s\;\in Z_{P^{*}}$ as its private key and calculates the public key *Y* = *s*.*P*. All these computations are completed off-line.

#### Registration Phase

A new user *U*_*i*_ such as a patient or health care professional purchases the smart card from the SP *and registers* it as follows:

Algorithm for registration:

Input: Password *pwd*_*i*_, nonce *r*, and ID.

Output: Encrypted personal information

**1:** *U*_*i*_ selects password *pwd*_*i*_ and a nonce *r*, and then *U*_*i*_ transfers the *ID* and computes

a. *A* = *h*(*pwd*_*i*_ ∥ *r*)

and sends it to the smart card *SP* along with an e-mail *e*_*i*_. Adding the nonce in the *pwd*_*i*_ ensures it does not reveal the sensitive information even to the smart card SP.

**2:** The smart card SP, after getting *ID, e*_*i*_, and A from the user *U*_*i*_, it computes

a. *M* = *h*(*s* ⊕*ID*)
b. B = *M* ⊕*A*

**3:** *SP* also finds the category *U*_*i*_ belongs to, such as patient or health care professional, and sends *{E*_*p*_*, P,Y,B, Category}* to *U*_*i*_, which it stores locally along with *r* in its smart cards.

**4:** The personal information that is entered by the SP is encrypted by the smart card using the public key Y and stored in it.

a. Smart card < - E_Y_(personal_info)

#### Login Phase

The login phase is common to patient and health care professional cards issued by the smart card SP.

*The user U*_*i*_ (patient or health care professional) logs in with the system to access the information stored in the smart card as follows:

**Algorithm for login:**

Input: ID and *pwd*_*i*_

Output: Login Access Request

**1:** *U*_*i*_ provides *ID* and *pwd*_*i*_, and then the smart card computes,

a. *A* = *h*(*pwd*_*i*_ ∥ *r*)
b. *M* = *B* ⊕ *A*
c. *C*1 = *a*.*P*
d. *C*2 = *a*.*Y*
e. *TID* = *ID* ⊕ *h*(*C*2)
f. *f* = *h*(*ID* ∥ *M* ∥ *T*)

Here, *a* is a secret nonce picked by *U*_*i*_, and *T* is the current time stamp.

**2:** *U*_*i*_ then transmits the login access request message

a. *msg*_1_ = {*C*1, *TID, f, T*} to SP.

#### Authentication Phase

The authentication steps are as follows: Algorithm for authentication

Input: Message msg_1_

Output: Unencrypted form of medical data

**1:** Upon receiving *msg*_1_ from *U*_*i*_, SP checks whether

a. *T*′−*T* ≤ Δ*T*

where Δ*T* is the maximum transmission delay. If the validity fails, SP rejects the session. Else, SP uses its private key *s* to compute

b. *C*2′ = *s*.*C*1
c. *ID*′ = *TID* ⊕ *h*1 (*C*2′)
d. *M*′ = *h*(*s* ⊕ *ID*′),
e. *f*′ = *h*(*ID*′ ∥ *M*′ ∥ *T*)

SP then checks whether *f*′ ? = *f*. If true, then *U*_*i*_, either the patient or health care professional, is a legitimate user. Else, abort.

**2:** *SP* then computes

a. *D*1 = *c*.*P*, *D*2 = *c*.*C*1

The session key

b. *SK* = *h*(*ID* ∥ *h*1(*D*2) ∥ *M*′) and
c. $G=h\left(SK∥M^{\prime }∥T_{2}\right)$

where, *c* is a nonce selected by *SP*, and *T*_2_ is the current time stamp. Then, *SP* transfers

d. *msg*_2_=*{D1,G,T*_2_*}* to *U*_*i*_.

**3:** Upon receiving *msg*_2_, *U*_*i*_ checks whether T_2_

a. $T^{\prime }-T_{2}\leq \;\Delta \;T\;$is valid or not. If it is valid, *U*_*i*_ calculates
b. *D*2′ = *a*.*D*1
c. *SK* = *h*(*ID* ∥ *h*1(*D*2′) ∥ *M*) for future correspondence.

**4:** After the mutual authentication, the current information of the patient from the smart card and the cloud server are fetched for proceeding with further treatment. In both cases, the patient's medical data are stored in the encrypted form. The data are fetched in the same form and then decrypted at the smart card to view in the unencrypted form by the health care professional.

#### Password Update Phase

*U*_*i*_ performs the following steps to update a password:

Algorithm for password update:

Input: ID and *pwd*_*i*_

Output: Updated password

**1:** *U*_*i*_ inputs *ID* and *pwd*_*i*_ and then calculates

a. *A* = *h*(*pwd*_*i*_ ∥ *r*)
b. *M* = *B* ⊕ *A*

**2:** Then, *U*_*i*_ is prompted to input the new password *pwd*_*new*_ and computes

a. *A*_*new*_ = *h*(*pwd*_*new*_ ∥ *r*)
b. *B*_*new*_ = *A*_*new*_ ⊕ *M*

and replaces *B* with *B*_*new*_; thus, the password is updated successfully.

According to the category, the health care professional belongs to the type of data retrieved, and data that is being synchronized with the cloud server differs. The below phases denote per the type of health care professional what type of data can be accessed or modified in the patient data.

#### Data Synchronization Phase

This phase starts when patient data is altered by the health care professional and should be synchronized with the cloud server to maintain consistency between the data stored in the cloud and the smart card and to store additional information that could not be stored in the cloud due to memory constraints. The following are the cases when the patient data should be synchronized with a cloud server.

- The doctor uploads an e-health prescription into the cloud after consulting with the patient and identify the health problem.
- When the doctor requires further diagnosis, the doctor refers the patient to the diagnosis center.
- From the diagnosis center, the test results are uploaded into the cloud database to help the doctor view the test results.
- The insurance provider can update the cloud database when the particular treatment bill is claimed.
- Similarly, additional health care professional information also can be stored in a cloud server.

After performing a successful login, the health care professional gets an option to store the information as follows:

**Algorithm: Data Synchronization**

Input: Msg_1_, different type of patient information

Output: Signature sig and encrypted file

**1:** The HCP defines the permissions for the patient document from the smart card used by the patient, for example, the pharmacist can view only the prescription information alone and that information is divided into chunks of byte arrays (B_1_*... B*_*n*_).

**2:** *HCP* then computes a. *MSG*_1_ = { *Category, scope*}⊕*SK*

*b. B*_1_= *B*_1_ ⊕ *SK*

*B*_*n*_ = *B*_*n*_ ⊕ *SK*

*c*. *MSG*_2_ = *h*(*ID* ∥ *Y*) ⊕ *SK*

**3:** *HCP* reconstitutes the file from byte arrays (*B*_1_*... B*_*n*_).

**4:** The *Category* is added to the file to denote whether the information pertains to the patient, doctor, insurer, or diagnostician. This help the cloud server while searching the different data according to their category.

a. If the category belongs to the patient, then it is further identified as to whether it is a prescription, test results, or insurance-related information.

**5:** HCP then computes an ECCDSA hash of the file to act as checksum and encrypts the file with its public key *Y* and sends the {Sig, F1,MSG2} to the cloud server S (48).

a. FD < -h(F),
b. Sig=S_y_ [FD]
c. *F1*=*E*_*y*_*[F]*

#### Data Retrieval Phase

This phase starts when patient data needs to be retrieved by the health care professional to study the patient's medical history and diagnose the disease to proceed with further treatment. The following are the cases when the patient data should be retrieved from the cloud server.

- The doctor wants to view the patient's previous history to know more in depth about the health problem.
- The pharmacist can sell the medicine according to the prescription uploaded in the cloud server.
- The insurance provider can check the hospital bill to process the claim for the medical expenditure.

**Algorithm: Data retrieval**

Input: Signature Sig, Encrypted file

Output: Unencrypted file

1: Data retrieval starts after the smart card authentication is over with the card issuer and request made for accessing additional information by the health professional.

2: After receiving the request, the cloud server S searches the data over the encrypted form from its database using the MCKS-MAT scheme (49). We have constructed the multiattribute tree (MAT) for the partient or health care professional record set by choosing the category as the root of the tree. File search is considered to be a separate phase.

3: The cloud server retrieves the data and then transfers it to the smart card SP. The decryption performed at the smart card solution provider by using s also computes and checks its ECCDSA hash to detect any tampering. If the check fails stop, divide the patient documents into chunks of byte arrays (B_1_... B_n_) and send it to the patient smart card

a. V=Ver_s_(Sig)
b. F=D_s_(F1)
c. (B_1_ ⊕ *SK*)…(B_n_ ⊕ *SK*_))_=Split(F)

*B*_1_ ⊕ *SK*

*B*_*n*_ ⊕ *SK*

4: The smartcard then decrypts the stream of messages and reconstitutes the file from byte arrays (B_1_... B_n_).

### File Search

To search the patient records in the cloud server, we adopt the MCKS-MAT scheme (49) by which we have constructed the MAT for the patient record set. The number of levels in the MAT index tree L is equal to the number of attributes in the patient file. The MAT index tree is encrypted using the ECC encryption algorithm. Along with the patient files, the encrypted MAT tree is stored in the cloud server, which protects the cloud server against a cipher text attack, known plaintext attack and known background attack. The construction and explanation of the MCKS-MAT scheme is beyond the scope of our work.

## Informal Analysis

We have assessed that the proposed method has the ability to protect the user from different cryptographic attacks.

### User Anonymity

Our scheme provides user anonymity, such as patient and health care professional (doctor, lab technician, and insurer) anonymity, for example, during the entirety of the phases, the user's ID is always masked and unattainable even from any trapped messages. Hence, the smart card SP, after getting *ID, e*_*i*_, and A from the user *U*_*i*_, it computes *M* = *h*(*s* ⊕*ID*) and B = *M* ⊕*A* and discloses it to the user. Furthermore, the ID is not revealed to anyone. Hence, our scheme confers the property of user anonymity.

### Forward Secrecy

Our scheme confers the forward secrecy property as each session key is fresh due to the randomness of *c*. *SP* computes *D*1 = *c*.*P*, *D*2 = *c*.*C*1. From that, it computes the session key *SK* = *h*(*ID* ∥ *h*1(*D*2) ∥ *M*′). Thus, each session key is completely autonomous of other sessions. Thus, even in the unlikely case that a session key is compromised, it does not affect other sessions.

### Replay Attack

Our scheme protects against replay attacks by providing sufficient checks of validity for each transmitted message. Thus, our scheme is able to withstand a replay attack as it includes the time stamp in the transmitted message. Kumar et al. (12) does not sustain doctor unlinkability. Kumar et al. (12, 23) does not support forward secrecy, stolen smart attacks. Our scheme protects against several known security attacks (50).

### Man-in-the-Middle Attack

Our scheme prevents the man-in-the-middle attack as each datum that we are transferring between the entities is associated with the time stamp and hash conditions. In case any adversary A verifies the time stamp, it further has to verify *f*′ = *f*, which is impossible due to the characterization of the one-way hash function.

### Data Confidentiality

In case any adversary tries to read the patient's or health care professional's information, it needs to decrypt the information, which is not possible without knowing the key and hash value. The freshness of s and one-way hash function ECCDSA ensures data confidentiality.

### Data Non-repudiation

The proposed protocol supports data non-repudiation in various phases. During the data synchronization phase, the signature is calculated as FD < -h(F), Sig=S_y_ [FD] and sends it to the cloud server along with the encrypted file { Sig, F1}. At the data retrieval phase, it verifies the signature as V=Ver_s_(Sig) by the smart card SP. This ensures that the authenticity cannot be denied by the health care professional.

### Patient and Doctor Unlinkability

Patient/doctor unlinkability means that adversary *E* should not reveal the medical association between the patient and the doctor *via* the communication channel. Because of the proposed protocol, both the patient's and doctor's information are stored in the encrypted form as *F1*=*E*_*y*_*[F]* and does not reveal the information even to the cloud server, so the unlinkability is preserved between the doctor and patient.

## Performance Analysis

We performed various cryptographic operations on a machine with a dual core processor of 2:4 GHz and equipped with 2 GB RAM running with the windows 10 operating system. Because of the other processes executing on the system, the execution time recollected in this article is the average time after a certain number of executions of the different cryptosystems based on article (51). The encryption and decryption of the ECC algorithm uses the key with the length of 163 bits. The security, communication, and execution cost of the proposed protocol with other relevant protocols are discussed in this article. In the following section, the security feature and communication and execution costs of the proposed protocol are compared with the Kumar et al. (12, 23) scheme. The evaluation made in this section delivers an effectiveness of the proposed protocol compared with the other relevant protocol.

The Li et al. (29) scheme does not support patient anonymity, patient unlinkability, doctor unlinkability message authentication, or session key security. It does not protect from the impersonation attack, stolen smart card attack, or off-line password guessing attack. Kumar et al. (12) does not support doctor unlinkability and forward secrecy. Kumar et al. (23) does not support forward secrecy. However, all the schemes do not support the stolen smart card attack as it is focusing on telecare medicine information system. In summary, our scheme provides support for several security features and protects against several known attacks.

### Computation Cost

In this section, we project performance of our proposed framework with the related schemes that operated in the cloud computing environment to enable secure medical data communication, such as the Kumar et al. and Li et al. techniques. Table 1 shows security feature comparison of various protocols. We have embraced different cryptographic operations in this article established on the details appropriate in Kumar et al. and Li et al. to assess the computation cost of the proposed protocol. Table 2 presents the execution time of various cryptographic operations, such as key generation, signature generation, ECC encryption/decryption, and symmetric cryptographic operations. Table 3 shows the computation cost of the SCB-HC protocol with other relevant protocols. The computation cost of the proposed protocol is 1.6169, which is slightly higher than the existing protocols. However, our scheme adopts the ECC cryptographic operations, Table 4 shows the communication cost of various components in bits which are not used in any of the existing schemes. Because it uses ECC operations, our scheme is more against the existing scheme. The efficiency of the proposed protocol in each phase is shown with other relevant protocols (52).

**Table 1:** Security feature comparison of various protocols.

**Security attack**	**Li et al. (29)**	**Kumar et al. (12)**	**Kumar et al. (23)**	**Proposed**
Man-in-the-middle attack	✓	✓	✓	✓
Replay attack	✓	✓	✓	✓
Patient anonymity	×	✓	✓	✓
Patient unlinkability	×	✓	✓	✓
Doctor unlinkability	×	×	✓	✓
Data non-repudiation	✓	✓	✓	✓
Data confidentiality	✓	✓	✓	✓
Message authentication	×	✓	✓	✓
Impersonation attack	×	✓	✓	✓
Stolen smart card attack	×	×	×	✓
Session key security	×	✓	✓	✓
Off-line password guessing attack	×	✓	✓	✓
Forward secrecy	×	×	×	✓

*✓ - Security attack protected by the protocols*.

*× - Security attack not protected by the protocols*.

**Table 2:** Execution time of various cryptographic operations.

**Notations**	**Descriptions**	**Execution time (s)**
*T*_*k*_*g*_ *en*_	Key generation time	0.219
*T*_*enc*_	ECC encryption time	0.3057
*T*_*dec*_	ECC decryption time	0.015
*T*_*k*_*g*_*en*1_	ECCDSA key generation time	0.466
*T*_*si*_*g*__*g*_ *en*_	Time for generating the signature	0.0009
*T*_*si*_*g*__*v*_ *erify*_	Time for verifying the signature	0.0053
*T*_*M*_	Time for performing multiplication.	0.0053 s
*T*_*H*_	Time for calculating one-way hash function	0.0005 s
*T*_*S*_	Time for calculating symmetric encryption/decryption time	0.0087 s

**Table 3:** Computation cost of SCB-HC protocol with relevant protocols.

**Phases**	**Li et al. (29)**	**Kumar et al. (12)**	**Kumar et al. (23)**	**Proposed**
Preliminary	NA	NA	NA	1*T*_*k*_*g*_*en*_ 0.219
Registration	3*T*_*H*_ 0.0015 s	3*T*_*H*_ 0.0015 s	3*T*_*H*_ 0.0015 s	2*T*_*H*_ + 1*T*_*Enc*_ 0.3067 s
HUP (health center data upload phase) (Login+ Authentication+ Data synchronization)	11*T*_*H*_ + 1*T*_*sign*_ + 3*T*_*s*_ 0.3543 s	10*T*_*H*_ + 1*T*_*sign*_ + 3*T*_*s*_ 0.3538 s	10*T*_*H*_ + 1*T*_*sign*_ + 5*T*_*s*_ 0.3628 s	12*T*_*H*_ + 1*T*_*si*_*g*__*gen*__ + 5*T*_*M*_ + 1*T*_*Enc*_ 0.339 s
TUP (treatment upload phase) (Login+ Authentication+ Data synchronization)	3*T*_*sign*_ + 6*T*_*s* + _10*T*_*H*_ 0.7128 s	2*T*_*sign*_ + 6*T*_*s* + _10*T*_*H*_ 0.7026 s	3*T*_*sign*_ + 6*T*_*s* + _11*T*_*H*_ 1.0528 s	12*T*_*H*_ + 1*T*_*si*_*g*__*gen*__ + 5*T*_*M*_ + 1*T*_*Enc*_ 0.339 s
MRP (medi reclaim phase) (Login+ Authentication+ Data Retrieval)	NA	NA	NA	12*T*_*H*_ + 1*T*_*si*_*g*__*ver*__ +5*T*_*M*_ + 1*T*_*Dec*_ 0.0528 s
LUP (lab technician phase) (Login+ Authentication+ Data Retrieval+ Data Synchronization)	NA	NA	NA	12*T*_*H*_ + 1*T*_*si*_*g*__*gen*__ + 1*T*_*si*_*g*__*ver*__ + 5*T*_*M*_ + 1*T*_*Dec*_ + 1*T*_*Enc*_ 0.3594 s
Password update	NA	NA	NA	2*T*_*H*_ = 0.001 s
Total time	1.0686 s	1.0579 s	1.4171 s	1.6169 s

**Table 4:** Communication cost of various components in bits.

**Component**	**Cost in bits**
Time stamp	48
Generated random number	48
Symmetric encryption/decryption operation	128
Asymmetric encryption/decryption operation	163
Modular multiplication and inverse operation	128
Cryptographic hash function	160
Executing/verifying a signature	256

**Figure 2:**
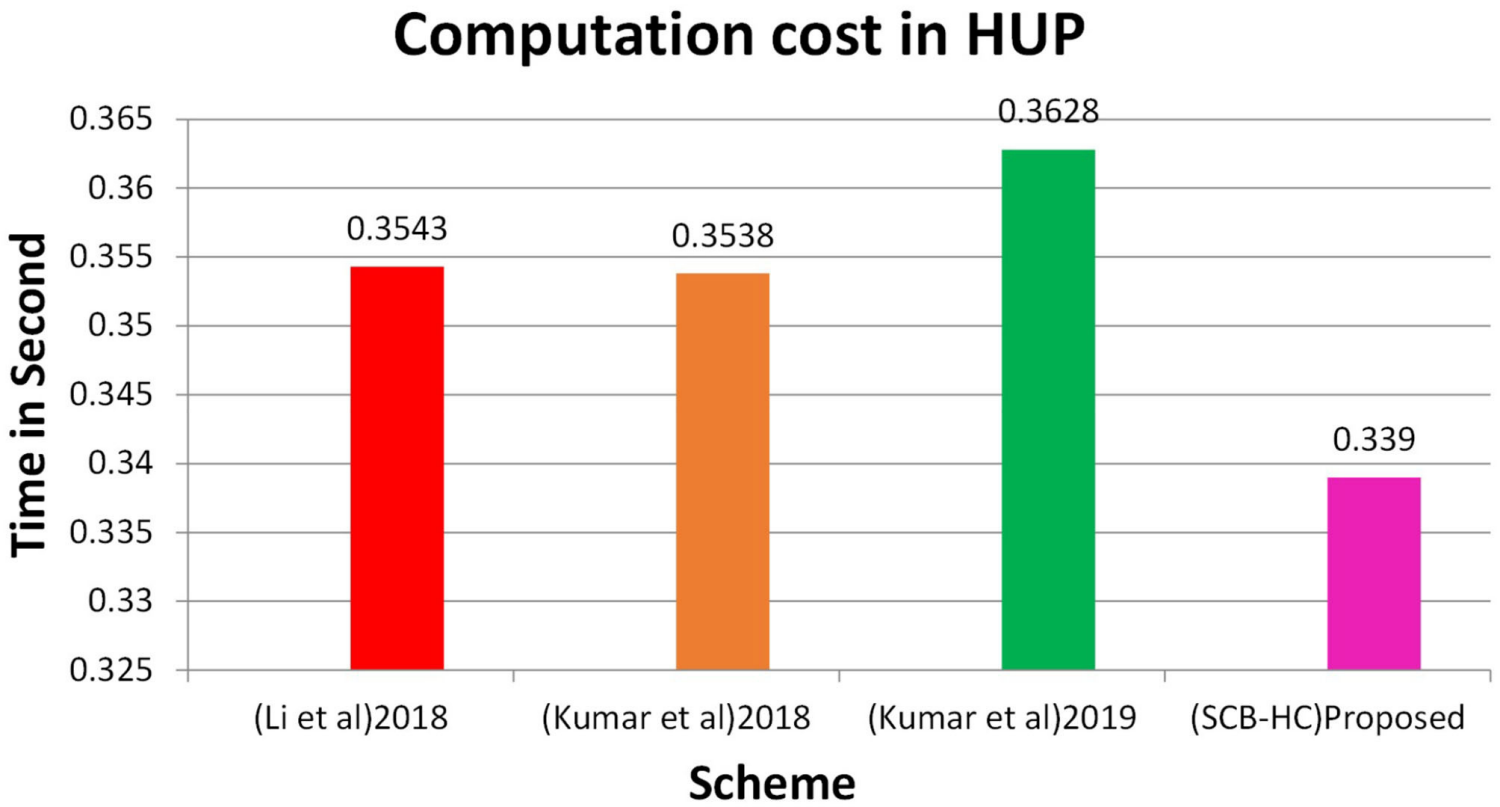
HUP computation cost.

**Figure 3:**
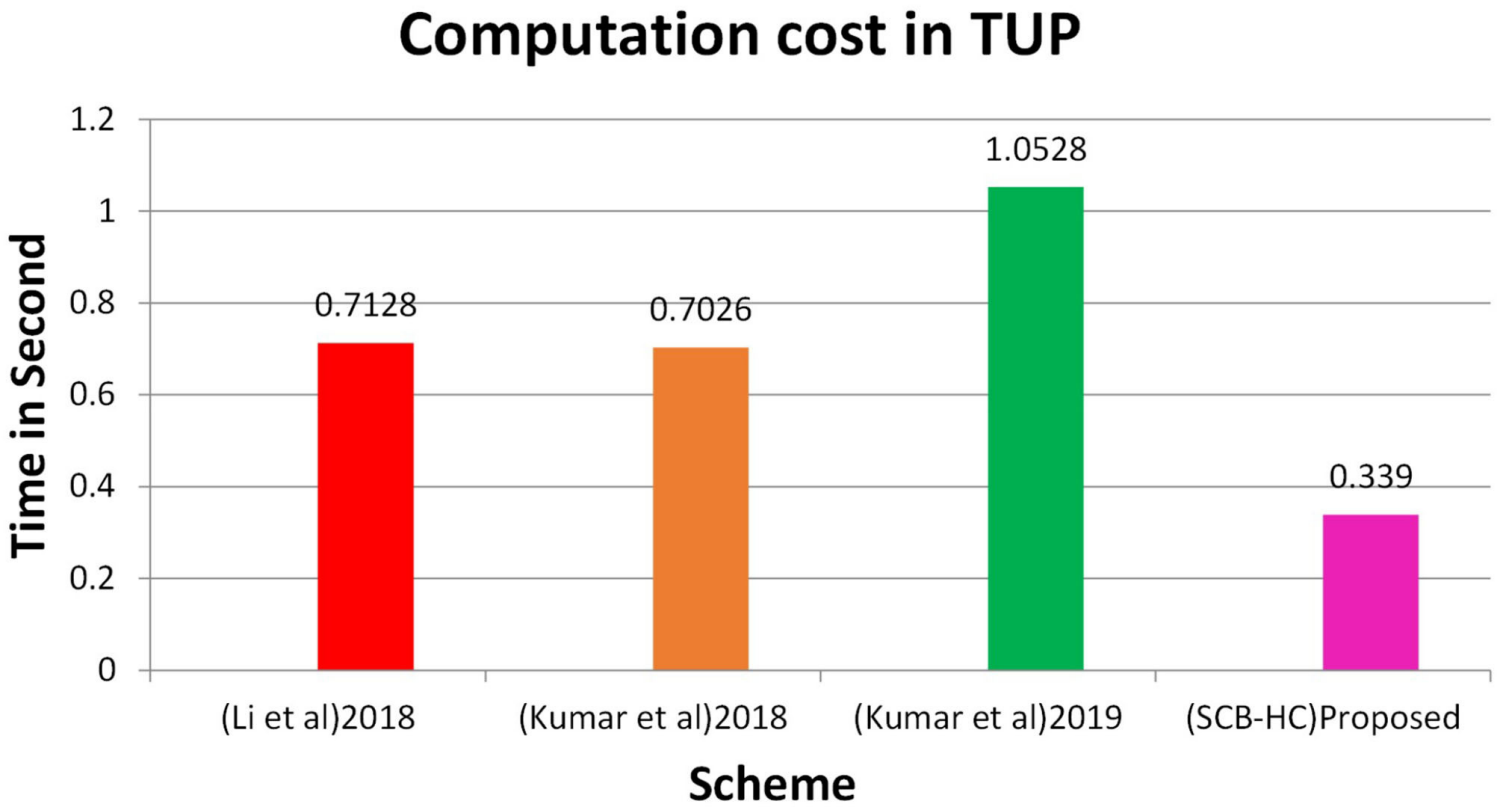
TUP computation cost.

**Figure 4:**
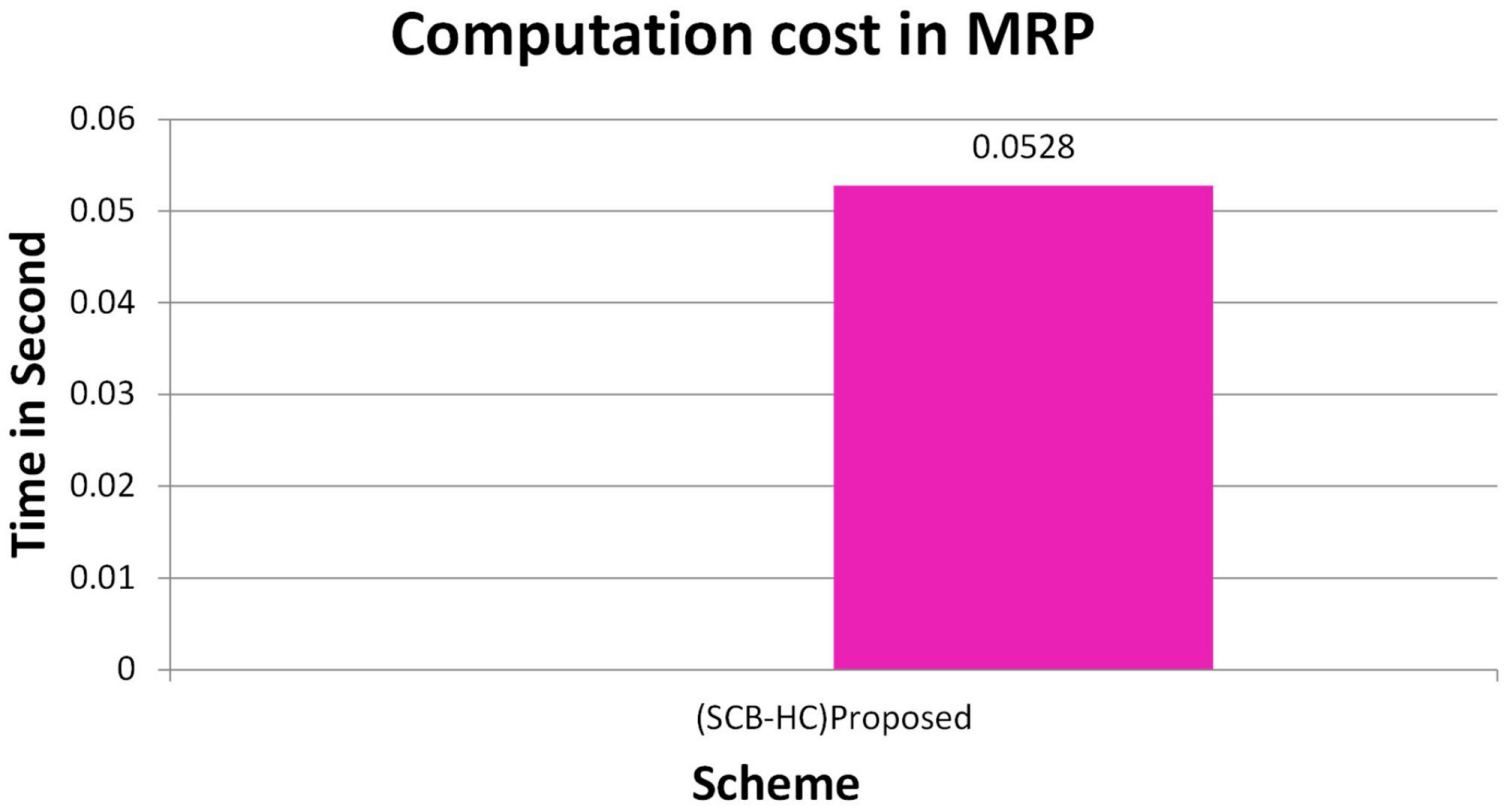
MRP computation cost.

**Figure 5:**
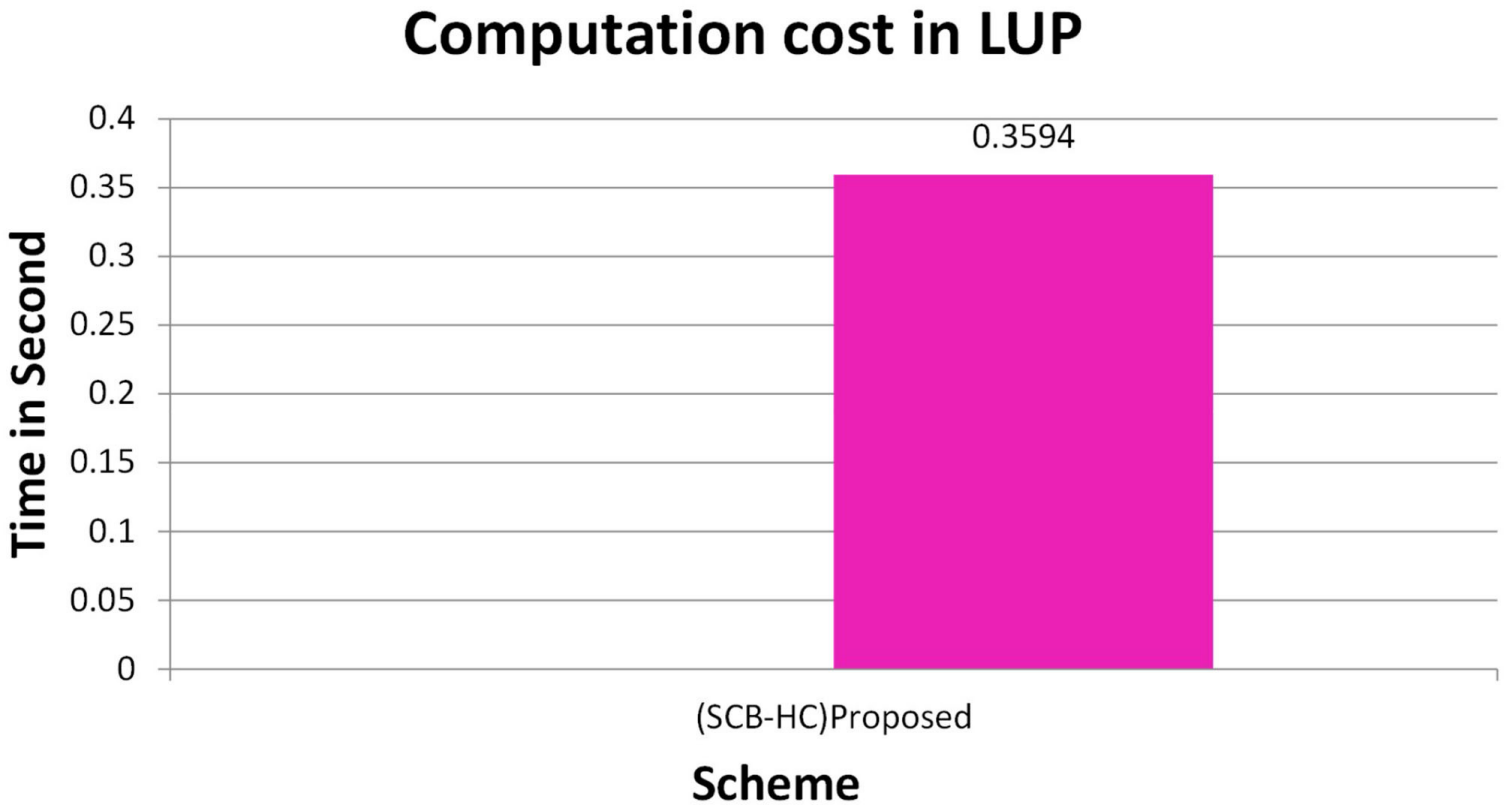
LUP computation cost.

**Figure 6:**
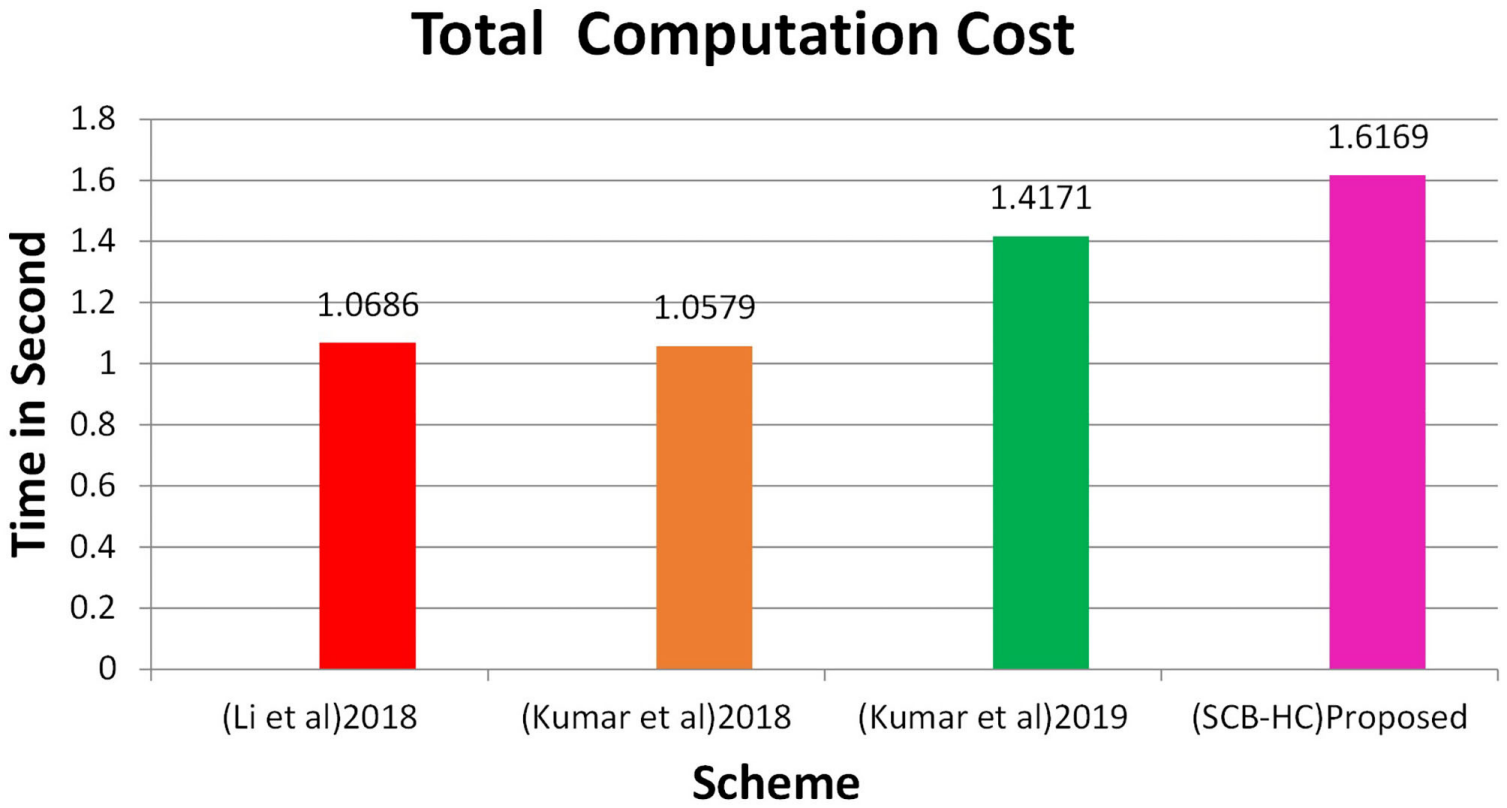
Total computation cost.

Figure 2 shows the computation cost in HUP in which our proposed scheme SCB-HC takes 0.339 seconds, which is less than the Li et al. (29) and Kumar et al. (12, 23) schemes even though our scheme SCB-HC is more secure compared with existing schemes. Figure 3 shows the computation cost of TUP. It also takes 0.339 seconds, which is less than the existing schemes. Figures 4, 5 shows the computation cost of MRP and LUP, which take 0.0528 and 0.3594, respectively. Only SCB-HC has this phase as it deals with the medi reclaim and lab technician upload phases, and this is the added advantage in the SCB-HC scheme that is not available in any of the existing phases. Figure 6 shows the total communication cost of all the scheme. The total cost of the SCB-HC scheme is 1.6169 s as it includes additional phases MRP and LUP, which are not present in any of the existing schemes. Also, SCB-HC uses ECC encryption and decryption and the ECCDSA signature algorithm, which is more secure and efficient compared with the existing schemes.

### Communication Cost Comparison

We analyze the communication cost of the SCB-HC protocol with other relevant protocols.

**Figure 7:**
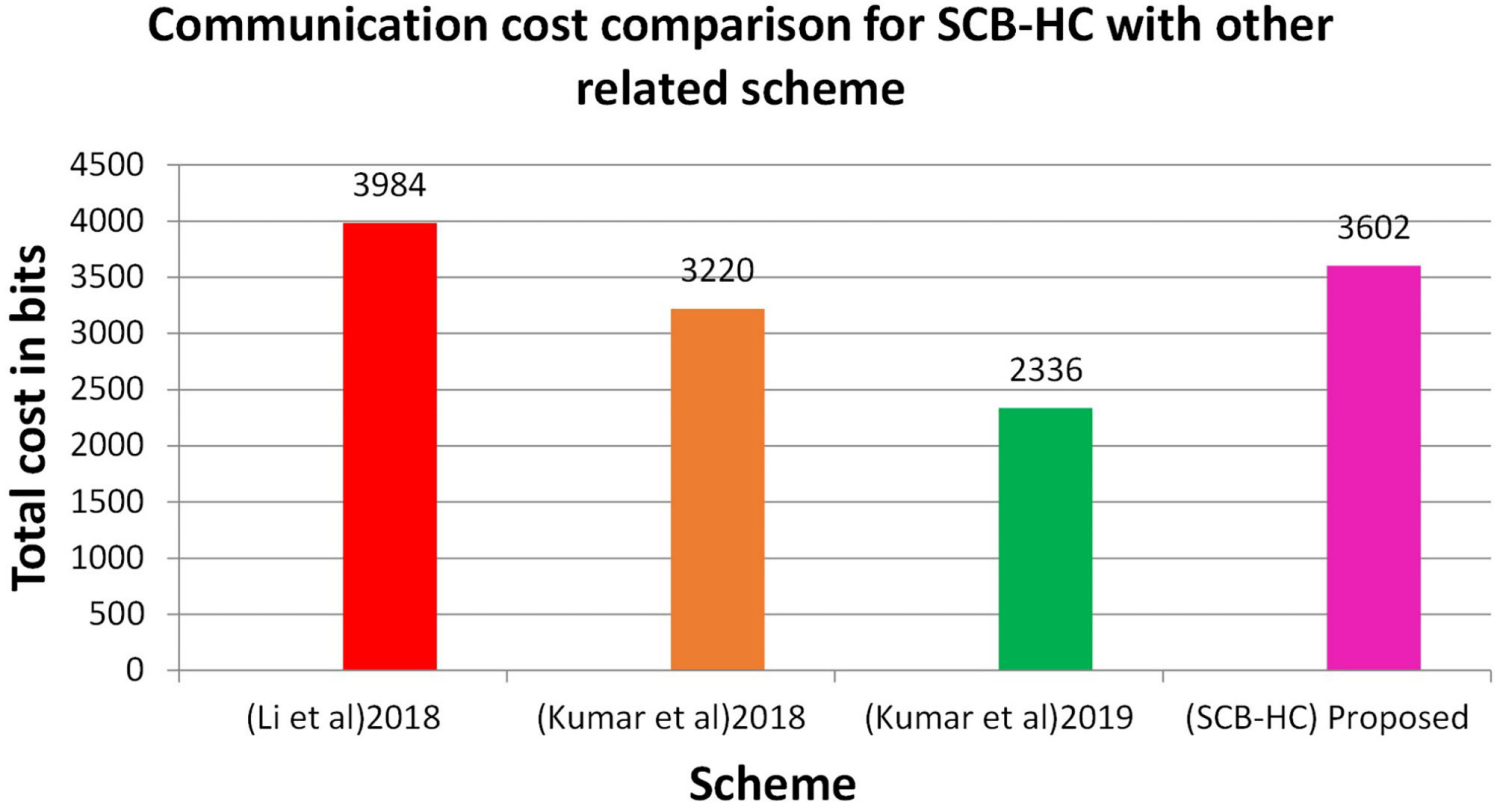
Communication cost for various schemes.

Table 5 shows the communication cost comparison of the various schemes in which SCB-HC has the communication cost of 3,602 bits and other relevant schemes are 3,984, 3,220, and 2,336 bits, respectively. Figure 7 shows the communication cost comparison for SCB-HC with various schemes. The SCB-HC has slightly higher cost compared with the Kumar et al. (12, 23) schemes as our scheme has various additional phases and the ECC cryptographic technique, which is not supported by other schemes.

**Table 5:** Communication cost comparison for SCB-HC with other related schemes.

**Protocol**	**Li et al. (29)**	**Kumar et al. (12)**	**Kumar et al. (23)**	**Proposed (SCB-HC)**
Preliminary	NA	NA	NA	211
Registration	208	208	208	371
HUP	592	624	496	755
TUP	720	544	544	755
MRP	NA	NA	NA	755
LUP	NA	NA	NA	755
PUP	1,232	544	496	NA
CP	1,232	596	592	NA
EP	NA	704	NA	NA
Total cost in bits	3,984	3,220	2,336	3,602

*HUP, Health center data upload phase; TUP, Treatment Upload phase; MRP, Medi reclaim phase; LUP, Lab Technician phase; PUP, Patient data upload phase; CP, Checkup Phase; EP, Emergency Phase*.

## Conclusion

We elaborated our suggested methodology for a remote authentication scheme with the smart card–based health care information using the ECC algorithm in the present article. We performed an informal analysis to substantiate the claim that our scheme provides sufficient security while maintaining usability. Maintaining user anonymity also maintains that others cannot access data without prior approval of the file owner. It also supports file integrity with the help of ECCDSA. Furthermore, we show that our proposed protocol is effective in terms of the communication and computation cost in secure cloud storage of smart card–based health care information.

The current work only involves integrating remote authentication with the smart card. In the future, we will focus on designing a smart card with a higher capacity to store large information, such as X-ray films and SCAN images.

## Data Availability Statement

The original contributions presented in the study are included in the article/supplementary material, further inquiries can be directed to the corresponding author/s.

## Author Contributions

SS and KB: conception or design of the work and data collection. SS, KB, and SB: data analysis, interpretation, and drafting the article. NK and GR: critical revision of the article and funding and final approval of the version to be published. All authors contributed to the article and approved the submitted version.

## Funding

This research was supported by the Faculty of Management of Comenius University in Bratislava, Slovakia.

## Conflict of Interest

The authors declare that the research was conducted in the absence of any commercial or financial relationships that could be construed as a potential conflict of interest.

## Publisher's Note

All claims expressed in this article are solely those of the authors and do not necessarily represent those of their affiliated organizations, or those of the publisher, the editors and the reviewers. Any product that may be evaluated in this article, or claim that may be made by its manufacturer, is not guaranteed or endorsed by the publisher.
